# Supplementary material for: The effect of arsenic on mitochondrial fatty acid metabolism via inhibition of carnitine palmitoyltransferase 1B and choline kinase beta in C2C12 cells
Source: PLoS One. 2025 May 29;20(5):e0320557. doi: 10.1371/journal.pone.0320557 (PMC12121733; doi:10.1371/journal.pone.0320557)
Supplement: Attachment — Submitted filename: Response to Reviewers.docx [file pone.0320557.s003.docx]

April 24, 2025

**Response to Editor:**

We thank the Reviewers for taking the time to review our manuscript and for their positive comments. We have revised our manuscript in accordance with the editor and reviewers’ suggestions, which resulted in much improved manuscript that easier to read and understand. Our responses to the comments are delineated below.

Journal Requirements:

When submitting your revision, we need you to address these additional requirements.

1. Please ensure that your manuscript meets PLOS ONE's style requirements, including those for file naming. The PLOS ONE style templates can be found at https://journals.plos.org/plosone/s/file?id=wjVg/PLOSOne_formatting_sample_main_body.pdf and https://journals.plos.org/plosone/s/file?id=ba62/PLOSOne_formatting_sample_title_authors_affiliations.pdf

**Response: We have verified that the manuscript complies with PLOS ONE’s formatting and file naming requirements.**

2. We note that the grant information you provided in the ‘Funding Information’ and ‘Financial Disclosure’ sections do not match.

When you resubmit, please ensure that you provide the correct grant numbers for the awards you received for your study in the ‘Funding Information’ section.

**Response: The grant information has been updated in the submission system.**

3. We note that your Data Availability Statement is currently as follows: All relevant data are within the manuscript and its Supporting Information files.

Please confirm at this time whether or not your submission contains all raw data required to replicate the results of your study. Authors must share the “minimal data set” for their submission. PLOS defines the minimal data set to consist of the data required to replicate all study findings reported in the article, as well as related metadata and methods (https://journals.plos.org/plosone/s/data-availability#loc-minimal-data-set-definition).

For example, authors should submit the following data:

- The values behind the means, standard deviations and other measures reported;

- The values used to build graphs;

- The points extracted from images for analysis.

Authors do not need to submit their entire data set if only a portion of the data was used in the reported study.

If your submission does not contain these data, please either upload them as Supporting Information files or deposit them to a stable, public repository and provide us with the relevant URLs, DOIs, or accession numbers. For a list of recommended repositories, please see https://journals.plos.org/plosone/s/recommended-repositories.

If there are ethical or legal restrictions on sharing a de-identified data set, please explain them in detail (e.g., data contain potentially sensitive information, data are owned by a third-party organization, etc.) and who has imposed them (e.g., an ethics committee). Please also provide contact information for a data access committee, ethics committee, or other institutional body to which data requests may be sent. If data are owned by a third party, please indicate how others may request data access.

**Response: The minimal raw dataset has been uploaded.**

4. Please review your reference list to ensure that it is complete and correct. If you have cited papers that have been retracted, please include the rationale for doing so in the manuscript text or remove these references and replace them with relevant current references. Any changes to the reference list should be mentioned in the rebuttal letter that accompanies your revised manuscript. If you need to cite a retracted article, indicate the article’s retracted status in the References list and include a citation and full reference for the retraction notice.

**Response: All the references listed are complete and correct.**

**Response to Reviewers:**

1. Is the manuscript technically sound, and do the data support the conclusions?

The manuscript must describe a technically sound piece of scientific research with data that supports the conclusions. Experiments must have been conducted rigorously, with appropriate controls, replication, and sample sizes. The conclusions must be drawn appropriately based on the data presented.

Reviewer #1: Yes

**Response: We thank the Reviewer for the positive comments.**

2. Has the statistical analysis been performed appropriately and rigorously?

Reviewer #1: I Don't Know

**Response: As described in the manuscript “Data analysis was performed using Origin 8.5 software (OriginLab, Northampton, MA) with one-way ANOVA followed by post hoc comparisons using the Holm-Sidak test. All data are presented as the mean ± standard error of the mean (SEM). A p-value of less than 0.05 (p < 0.05) was considered statistically significant.”**

3. Have the authors made all data underlying the findings in their manuscript fully available?

The PLOS Data policy requires authors to make all data underlying the findings described in their manuscript fully available without restriction, with rare exception (please refer to the Data Availability Statement in the manuscript PDF file). The data should be provided as part of the manuscript or its supporting information or deposited to a public repository. For example, in addition to summary statistics, the data points behind means, medians and variance measures should be available. If there are restrictions on publicly sharing data—e.g. participant privacy or use of data from a third party—those must be specified.

Reviewer #1: No

**Response: The minimal raw data set was uploaded.**

4. Is the manuscript presented in an intelligible fashion and written in standard English?

PLOS ONE does not copyedit accepted manuscripts, so the language in submitted articles must be clear, correct, and unambiguous. Any typographical or grammatical errors should be corrected at revision, so please note any specific errors here.

Reviewer #1: Yes

**Response: We thank the reviewer for the comment.**

5. Review Comments to the Author

Please use the space provided to explain your answers to the questions above. You may also include additional comments for the author, including concerns about dual publication, research ethics, or publication ethics. (Please upload your review as an attachment if it exceeds 20,000 characters)

**Reviewer #1:**

The study makes a valuable contribution to the understanding of arsenic-induced muscle toxicity and is suitable for publication after minor revisions. Here are a few suggestions before proceeding to publishing:

**Abstract** is effectively summarizing the study which addresses a relevant mechanism of arsenic toxicity in skeletal muscle.

1. Mentioning the used analytical methods maybe better for scientific rigor.

**Response: We thank the reviewer for the thoughtful suggestion. The analytical methods have now been thoroughly described in the 'Materials and Methods' section of the manuscript.**

2. Including a few representative findings such as % decrease in membrane potential, or fold change would add impact.

**Response: We would like to thank the reviewers for the constructive comments. Some significant decreasing, including the percent of the decreased membrane potential was added in the revised manuscript (lines 35-38).**

**Introduction** is well structured and well referenced. It builds a brief scientific rationale.

3. The aim of the study statement is given; however, the paper would benefit from a hypothesis statement to give a rather clear and mechanistic model for the study.

**Response: We thank the reviewer for the constructive suggestions. The hypothesis statement was added to the revised manuscript (lines 82-85).**

**Materials and methods:**

4. Seeding density for each test is important and should be given.

**Response: Seeding density was given in the revised manuscript (line 96).**

5. The sample size, biological and technical replicates were not mentioned in none of the experiments, and should be given

**Response: The sample size, along with the biological and technical replicates, has been described in the manuscript (lines 104, 117, 132, 135, 150, and 153).**

6. Reverse transcription of mRNA was not included. It is better to be included, in terms of mentioning the initial mRNA amount for following qPCR analysis.

**Response: The protocol of mRNA reverse transcription has been detailed in the revised manuscript (Lines 156-160).**

**Results:**

7. Phrases like “significantly impacted” or “significantly inhibited” (e.g., Lines 170, 172, 184 and 222) should be accompanied by specific p-values and a statistical method reference. This could be given in the manuscript or in the figure legends.

**Response: The p-values were added to the manuscript (lines 204, 215, 224 and 230).**

8. The results mention decreases or inhibition, but the magnitude of changes is often not described.

**Response: The percentage of decrease or inhibition was added to the results (lines 178,181,196, 207, 216, 225, 231 and 232)**

**Discussion:**

9. Condensing some sections which are already given in the results (e.g. line 227- 237, lines 303 – 309.)

**Response: The correction is reflected in lines 237–240 and lines 306–314.**

10. Given the relevance to human exposure and environmental health, it would be useful to include a brief comment on how these findings could be translated.

**Response: A brief comment on how our findings contribute to understanding the mechanism of arsenite-induced damage in human skeletal muscle is provided in lines 341–343.**

11. What a decreased fatty acid biosynthesis related to CHKB suppression might impact mitochondrial membrane stability can be further discussed.

**Response: The further discussion on the impact of decreased fatty acid biosynthesis related to CHKB suppression on mitochondrial membrane stability was added in lines 313-314.**

12. At the very end of the last paragraph, future directions would better be given.

The manuscript is intelligible and written in standard English but would benefit from minor language editing to enhance clarity and polish the overall presentation. Some of the occasional awkward phrasings and punctuation errors are stand out.

**Response: The future direction was given on the line 344.**

6. PLOS authors have the option to publish the peer review history of their article (what does this mean?). If published, this will include your full peer review and any attached files.

If you choose “no”, your identity will remain anonymous, but your review may still be made public.

Do you want your identity to be public for this peer review? For information about this choice, including consent withdrawal, please see our Privacy Policy.

Reviewer #1: No

Sincerely,

**Xian Wu**, PhD

Department of Pharmacology and Toxicology

Brody School of Medicine,

East Carolina University

Greenville, N.C. 27834

USA

**Yan Gao**, MD

Department of Cardiovascular Medicine

Xi’an Trade Union Hospital

Xi’an, Shaanxi Province, 710199

China
